# Small group gender ratios impact biology class performance and peer evaluations

**DOI:** 10.1371/journal.pone.0195129

**Published:** 2018-04-03

**Authors:** Lauren L. Sullivan, Cissy J. Ballen, Sehoya Cotner

**Affiliations:** 1 Department of Ecology, Evolution and Behavior, University of Minnesota, Saint Paul, MN, United States of America; 2 Department of Biology Teaching and Learning, University of Minnesota, Minneapolis, MN, United States of America; University of Westminster, UNITED KINGDOM

## Abstract

Women are underrepresented in science, technology, engineering, and mathematics (STEM) disciplines. Evidence suggests the microclimate of the classroom is an important factor influencing female course grades and interest, which encourages retention of women in STEM fields. Here, we test whether the gender composition of small (8–9 person) learning groups impacts course performance, sense of social belonging, and intragroup peer evaluations of intellectual contributions. Across two undergraduate active learning courses in introductory biology, we manipulated the classroom microclimate by varying the gender ratios of learning groups, ranging from 0% female to 100% female. We found that as the percent of women in groups increased, so did overall course performance for all students, regardless of gender. Additionally, women assigned higher peer- evaluations in groups with more women than groups with less women. Our work demonstrates an added benefit of the retention of women in STEM: increased performance for all, and positive peer perceptions for women.

## Introduction

The attrition of women in science, technology, engineering, and mathematics (STEM) disciplines is an issue of global concern [1], attracting considerable interest from educators, administrators, and policy makers [2]. Explanations for female attrition in STEM degrees and careers are complex [3,4], and include intangibles like gender bias and science confidence [5–7], which can be difficult to study with empirical research or address with scalable interventions. Recent evidence suggests that biases about gender and intellectual ability form as early as 6 years of age [8], and these STEM-related biases continue to perpetuate as students age. At the undergraduate level, women report lower science confidence [9], imposter syndrome [10], and higher susceptibility to stereotype threat [11,12]. Even the field of biology, which tends to recruit and retain far more female students than other STEM fields [13,14], shows lower achievement and participation of undergraduate female students as compared to their male counterparts [15]. As the need for trained workers with STEM undergraduate degrees increases [16], clarifying and promoting factors that decrease the gender gap in STEM will contribute to meeting national worker needs and promote overall gender equality (e.g. lowering the wage gap [17–19]).

The gender gap is particularly pronounced in large introductory gateway courses at the University level that are known for ‘weeding out’ students who are ‘not cut out for’ a particular discipline [20]. Gateway courses have a disproportionately negative effect on women, who are more likely than men to leave the major if they receive low grades [21]. When faced with a ‘chilly [classroom] climate’—typically in large classroom settings where instructors unknowingly bias their efforts toward male students [22,23], female students tend to feel less engaged in discussions, and perceive themselves to be engaged less often by instructors [24]. In such environments, a sense of inclusion plays an important role for increasing retention of students in STEM disciplines [25]. This may be particularly true for women in STEM who report feeling a lower sense of belonging [26] and higher levels of discrimination in STEM, as compared to women in more female-dominated academic areas [27]. Understanding the mechanisms that decrease the gender gap in gateway courses is necessary to increase the number of women with STEM degrees.

One approach to diversify introductory STEM courses is through the local classroom environment. For example, exposing students to instructor role models [9], or removing gendered visual signs in learning spaces [28] promote female confidence and interest in the subject matter to the level of their male peers. Here we propose another solution: that altering the classroom microclimate (i.e. the makeup of small learning groups) can positively influence female students. Specifically, we hypothesize that learning groups with female-majority gender ratios will positively influence quantitative performance and evaluative metrics for women in biology. Recent work demonstrates that altering the gender ratio of small working groups can influence both instructor and student attitudes. Female-majority gender ratios in groups increases intention to pursue STEM and eagerness to complete STEM tasks for women, especially for first year students [29]. However, other work demonstrates that more women in groups can lead to negative intragroup perceptions or negative ratings from external evaluators [30,31]. By understanding how small group gender ratios influence students, instructors can consider the role of social dynamics, while making informed decisions about how to structure their own class.

Here, we determined how the gender ratio of small groups influenced student learning in introductory biology courses for non-biology majors. We analyzed the effects of gender ratios within three large introductory biology classrooms (average classroom size is approximately 130 students) by either deliberately or randomly assigning students to groups and then fixing their seats for the remainder of the semester. We quantified the following: (1) student academic performance; (2) self-reported sense of social belonging; (3) peer- and self-evaluations of the extent to which students participated in their group, and the quality of those contributions.

## Methods

### In-class group work

Our study focused on three sections of two introductory biology courses for non-biology majors, BIOL 1003 (instructor A, section 1, *N* = 114, and section 2, *N* = 116) and BIOL 1050 (instructor B, section 1, *N* = 161). The courses were designed to address general biological principles including scientific inquiry, history of evolutionary thought, principles of genetics, the nature of the nature of variation, behavioral ecology, human evolution and human population growth. The courses had two midterm exams and one final exam that accounted for 41% of the final course grade. The remainder of students’ grades were composed of in-class lecture quizzes (7% of course grade; best 10 of 11 quizzes over the semester), in-class lecture assignments and participation (19% of the course grade; rewarded collaborative group work and in-class contributions), and the laboratory component of the course (33% of the final course grade).

**Fig 1:**
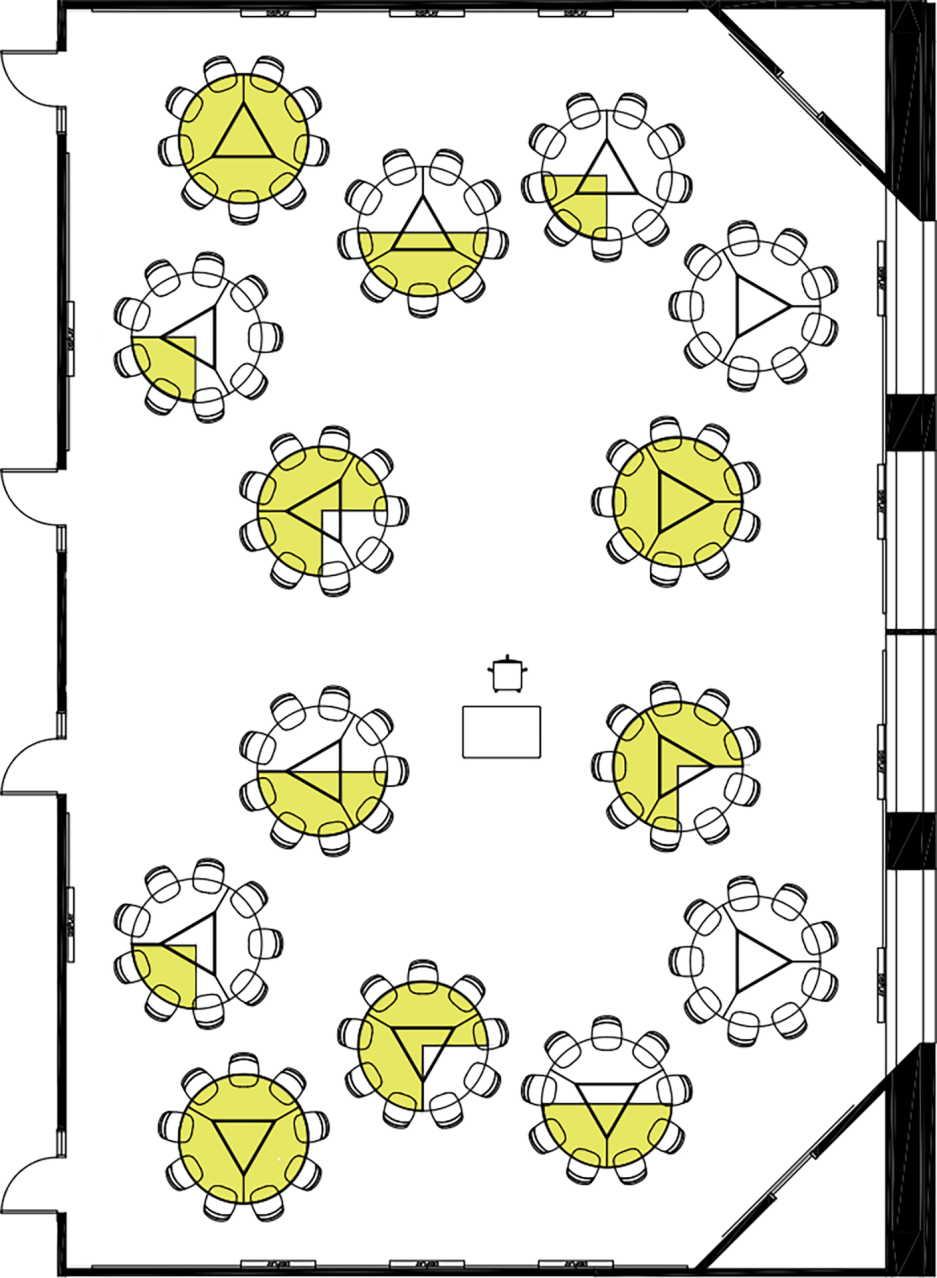
Deliberately manipulated student gender ratio at each table in large active learning classrooms in Bruininks Hall at the University of Minnesota. Yellow depicts the proportion of women we assigned to each table for the entirety of the fall 2016 semester: all women (100% women), women majority (75% women), gender parity (50% women), women minority (25% women), or all men (0% women). Image modified from the University of Minnesota office of classroom management (www.classroom.umn.edu).

All three sections were taught in active learning classrooms at the University of Minnesota [32]. In BIOL 1003, students were assigned a table where they sat for the duration of the semester. Students in this sample were not biology majors and few were STEM majors (i.e., 5% of students belonged in a STEM college at the University of Minnesota). We designed each table to have a gender ratio of all women (100% women), women majority (75% women), gender parity (50% women), women minority (25% women), or all men (0% women; Fig 1). In BIOL 1050, students were randomly assigned to sit at a numbered table for the duration of the semester, and we obtained post-hoc gender ratios from these randomly assigned groups. We collected performance information (grades) from all three classes, and peer-evaluation information from BIOL 1003 only.

Instructors used active learning pedagogy in all three classes for the duration of the semester, and thus students had ample opportunity to interact with one another and develop perceptions of their peers and their own performance in their group. Active learning pedagogies included: 1) student groups working on structured assignments in which students explained their reasoning and worked together to solve problems during lecture; 2) personal response systems (‘clickers’) used for graded in-class multiple choice questions that students could discuss with their group; 3) point allocation that rewarded group work and ongoing preparation rather than exam performance exclusively. Based on observing 16 classes of BIOL 1003 section 1, the instructor interacted with students an average of 14.5 times over the 1 hour 15 minute course period. In BIOL 1003 section 2, the instructor interacted with students an average of 17 times over a class period. And in BIOL 1050, the instructor interacted with students an average of 19.5 times over the 1 hour 15 minute course period. Therefore, by using student-instructor interactions as a proxy for active learning, each lecture consistently used in-class activities.

### Data collection

The instructors provided student grades, which were matched to student institutional information, including gender, and de-identified prior to analysis. We relied on institutional gender data that does not necessarily reflect the complexity of gender identity. Students took the peer- and self-evaluation survey at the midway point and end of the course to gauge student perceptions of their group members and themselves over the entire semester. Both peer- and self-evaluations contained the same questions. The evaluations asked to what extent students agreed with the following statements about their peers or themselves: 1) this group member regularly shows up to class, 2) this group member regularly contributes ideas and suggestions during group discussions, and 3) this group member exhibits a strong understanding of course material (S1 Appendix). The scale was coded as follows: 1 = strongly disagree, 2 = disagree, 3 = neutral, 4 = agree, 5 = strongly agree. We conducted a factor analysis on students’ self-evaluation to explore whether these data were suitable for factor reduction. In this case, we found the Kaiser-Meyer-Olkin (KMO) Measure of Sampling Adequacy for the whole dataset was KMO = 0.710 and Bartlett's test of sphericity was p < 0.001. The single reduced component explained 81% of the total variance. We tested for internal consistency using Cronbach’s alpha, and found them to be highly correlated (Cronbach’s alpha > 0.8). In response to this analysis, we used an average of the survey items, representing one measure of evaluation.

To examine sense of social belonging of students, we used four survey questions modified from Cornell University’s Student Engagement and Inclusion Survey (for more information see http://irp.dpb.cornell.edu; S2 Appendix); these responses were also quantified on a 5-point Likert scale. We asked students to what extent they agree or disagree with statements related to classroom and university social belonging. All students took this survey only once at the end of the course because it was designed to gauge social belonging over the entire semester. We conducted a factor analysis to explore whether these data were suitable for factor reduction. For the four items, we found the Kaiser-Meyer-Olkin (KMO) Measure of Sampling Adequacy for the whole dataset was KMO = 0.783 and Bartlett's test of sphericity was p < 0.001. A reduced single component explained 65% of the total variance and Cronbach’s alpha > 0.8. We used an average of the survey items, representing one measure of social belonging.

All methods of data collection in this study, along with students’ written informed consent, was approved through the University of Minnesota Institutional Review Board (IRB 1405E50826). All student participants were above the age of 18.

### Statistical analyses

We performed all statistical analyses in R v3.3.2, an open-source statistical programming language [33]. We analyzed both performance and student evaluation data using linear mixed effects models with the lmer() function in the lme4 package [34], with the addition of the lmerTest package [35] to produce p values. All data and code can be found in the supplemental information (S1–S3 Data, S3 Appendix).

### Performance

To analyze student performance, our dependent variable was calculated as a z-score of student’s final grade ((student’s final grade–mean class final grade) / standard deviation of class final grade) in order to determine how student performance was affected relative to class mean grade. A student’s final grade included their performance on three exams, in-lecture quizzes, in-class lecture assignments, participation, and the laboratory component. For this analysis, we used student course performance data from both BIOL 1003 and 1050, for a total of 377 students. Fixed effects included an interactive model with student gender, the percent female makeup of their classroom group, as well as student’s composite ACT/SAT score. Random effects included classroom group identity nested in lecture section nested in course.

### Social belonging

We examined student reported sense of social belonging using a mixed effects model with the averaged social belonging score (described above) as the dependent variable. Again, fixed effects included the interaction of gender and percent female in group, while random effects included learning groups nested within lecture section, nested within course. This analysis included 268 students.

### Peer- and self- evaluation

To evaluate how student peer- and self-evaluation scores were influenced by an individual’s gender and their small group’s gender ratio, we ran a model where our dependent variable was the overall mean score of three evaluative statements from evaluators (assigned to peers or themselves). This analysis included 238 students. Fixed effects included the interactive effects of evaluator gender, the percent of the small group that was female, and if students were evaluating themselves or others. The peer- and self-evaluation was only given to students in the BIOL 1003 class, thus the random effects for this analysis included classroom group nested within lecture section.

## Results

### Performance

**Fig 2:**
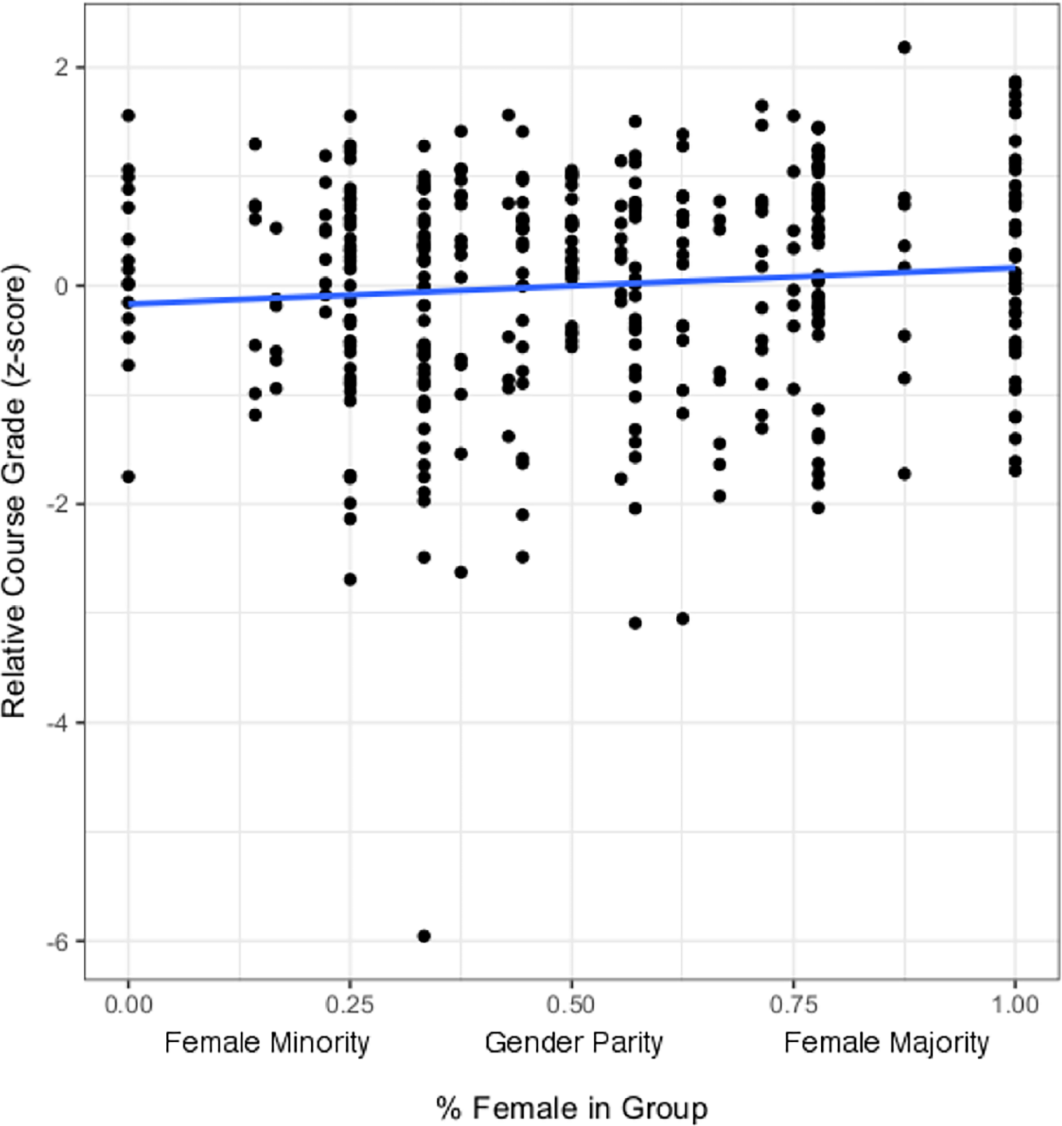
Relative course grade in relation to the gender ratio of classroom groups. As the percent of females in each group increase, so does relative course grade regardless of gender.

We found a non-significant interaction between student gender and the group gender ratio for classroom performance scores, thus we removed the interaction from the model. The main effect of group gender ratio did significantly predict performance (Fig 2, Table 1A); as the percent of women in each group increased, student performance increased relative to the classroom mean (p = 0.0258), regardless of gender (p > 0.05). As predicted [36], students’ combined ACT score also significantly predicted their course performance. We encourage teachers to transform their data to z-scores for comparison.

**Table 1:** Results of the statistical mixed effects models to explore how a) standardized classroom performance was influenced by student gender and the gender ratios in small groups, b) how students’ sense of belonging was affected by a students’ gender and the gender ratio of their small group, and how students’ final peer evaluation scores differ by the gender of the evaluator, the gender ratio of their small group, and if they are evaluating themselves or others in the c) full model, or a d) simpler model examining how female students evaluate their peers or e) themselves in relation to the group gender ratio.

**a)**	**Performance**					
		Estimate	Std. Error	df	t-value	p-value
	Evaluator Gender (M)	-0.081	0.109	290.99	-0.739	0.4605
	Percent Female	0.467	0.208	223.73	2.244	**0.0258**
	ACT Score	0.137	0.013	271.53	10.661	**<0.0001**
**b)**	**Sense of Belonging**					
		Estimate	Std. Error	df	t-value	p-value
	Evaluator Gender (M)	-0.193	0.107	243.66	-1.807	0.0720
	Percent Female	0.035	0.22	88.08	0.159	0.8740
**c)**	**Final Peer Eval.—full model**					
		Estimate	Std. Error	df	t-value	p-value
	Evaluator Gender (M)	0.304	0.063	606.10	4.838	**<0.0001**
	Percent Female	0.328	0.171	36.50	1.915	0.0634
	Type of evaluation (self)	0.412	0.059	598.20	6.955	**<0.0001**
**d)**	**Final Peer Eval.–Female only, peer model**					
		Estimate	Std. Error	df	t-value	p-value
	Percent Female	0.676	0.294	26.29	2.301	**0.0295**
**e)**	**Final Peer Eval.–Female only, self model**					
		Estimate	Std. Error	df	t-value	p-value
	Percent Female	0.031	0.242	36.78	0.128	0.8990

### Social belonging

**Fig 3:**
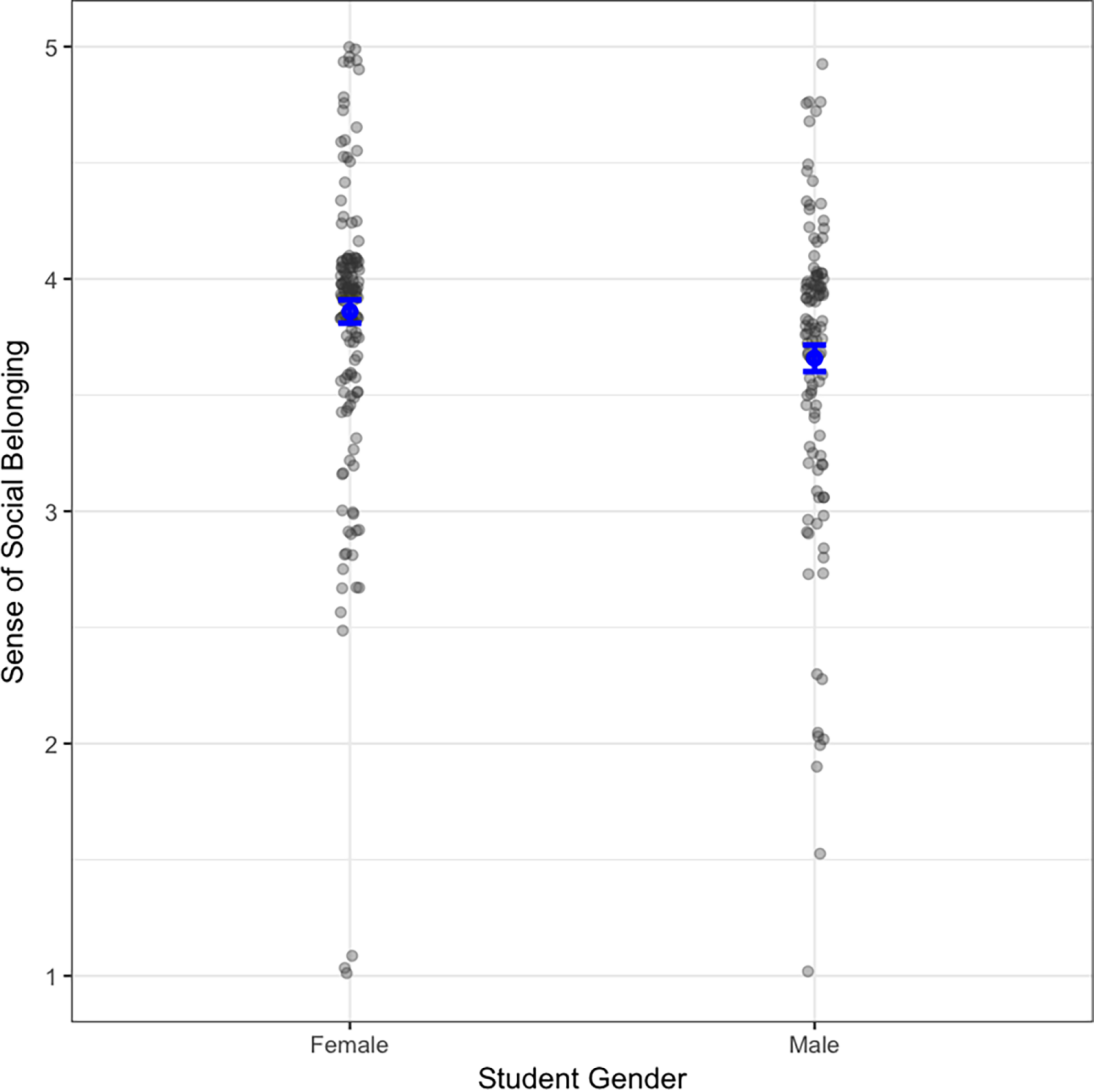
Students’ sense of social belonging in BIOL 1003 and 1050 classrooms. There was no statistical difference between men and women. Gray points represent all data, while blue points represent the mean and standard error of the data.

We found no significant interaction between gender and the percent of women in learning groups, and when we employed an additive model, we found no significant relationships (Fig 3, Table 1B). We do observe a trend that suggests gender influences sense of social belonging in BIOL 1003 and 1050 classrooms (Fig 3; p = 0.072), with women reporting higher belonging in the classroom. Future work will clarify what aspects of these classes created the inclusive climate for female students.

### Peer- and self- evaluation

**Fig 4:**
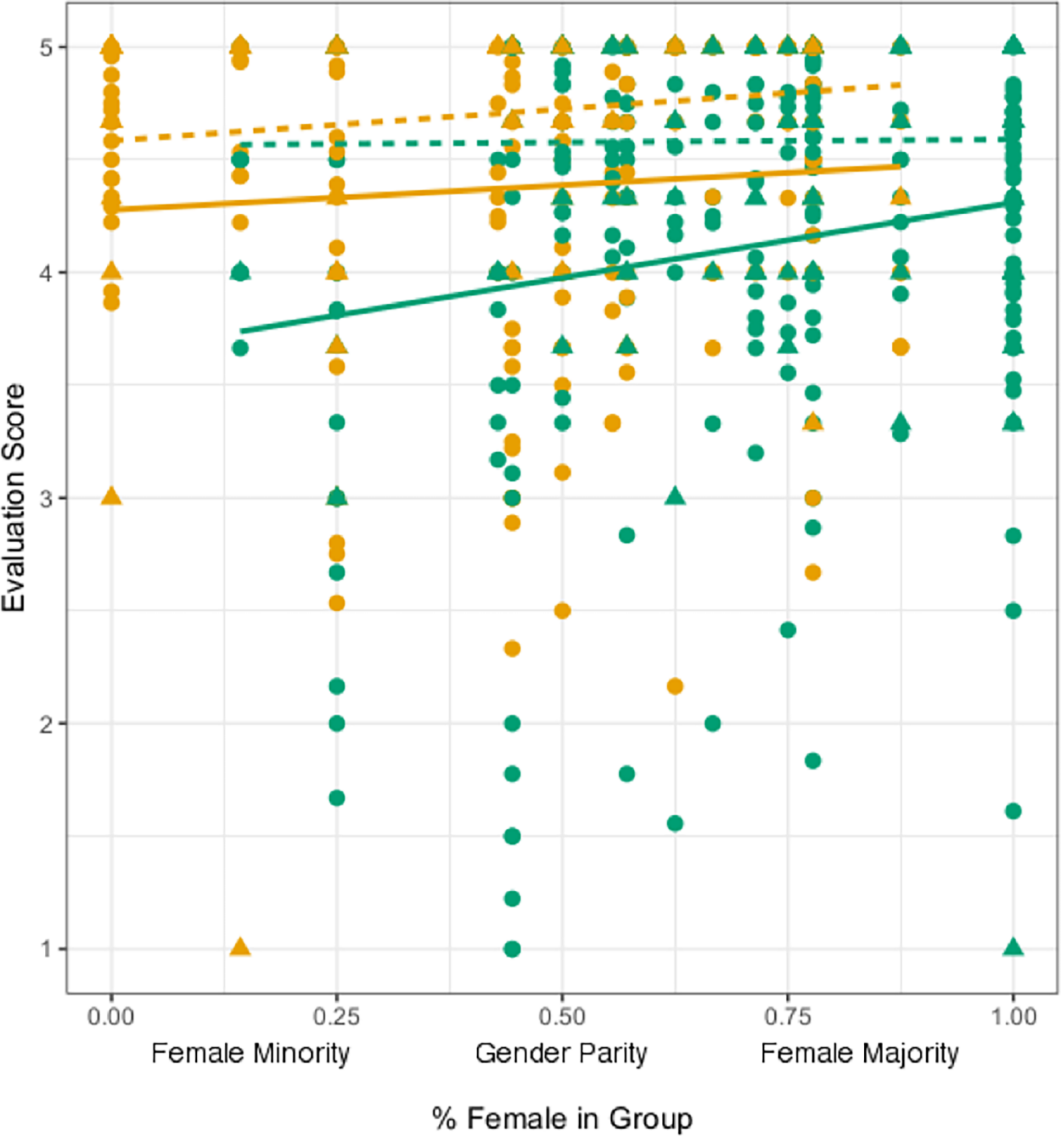
Average student evaluation scores per individual for themselves and others based on the gender of the evaluator. Triangles are self-evaluations (dotted lines) and circles are evaluations of others (solid lines), while green symbols are evaluations given by females and yellow symbols are evaluations given by males. In general, women tend to evaluate their colleagues higher as the percent of women in each group increases, but this is not true for men. In general, women tend to score more harshly than men, and everyone scores themselves higher than they score others.

For the midterm evaluation, we only found a significant effect of “self”; students evaluated themselves higher than they did others (p < 0.0001). We saw quite different results for the final peer- and self- evaluation. We did not find any significant interactions for the peer- and self- evaluation scores, however the additive model showed gender played a strong role in how students evaluated themselves and peers in their groups (Fig 4, Table 1C). First, all students tended to evaluate themselves higher than they evaluated others (p < 0.0001). Additionally, men provide overall higher evaluations for themselves and for others than women (p < 0.0001). Finally, when looking at the full additive model, while not statistically significant, self and peer evaluations tended to increase as the percent of women in the classroom group increased (p = 0.0634). Because we were interested in women specifically, we kept the random effects the same, but subset the data to isolate only female evaluations of themselves and others. We found that for women, group gender ratio significantly predicted peer evaluations, with women assigning higher grades to their peers as the percent of women in the group increased (p = 0.0295, Table 1D). Using this model structure, however, there no significant relationship between group gender ratio and self-evaluation scores (p = 0.899, Table 1E).

## Discussion

We show that the classroom microclimate had a significant impact on the learning environment for students in three large introductory biology classrooms. Female-majority groups had a significant, positive influence on student performance regardless of gender, and women’s peer- evaluations across three active learning classrooms. This result indicates the importance of retaining women in STEM disciplines, as the inclusion of women may accomplish more than simply addressing gender diversity. We found that overall, women judged themselves and others more critically than men, which has also been found in other peer evaluation studies [37]. We did not find that gender ratio manipulations influenced students’ sense of social belonging.

Our analyses support the hypothesis that the presence of women positively influences students. Increasing the percent of females in a group can lead to positive in-class affective outcomes for women such as decreased anxiety and increased confidence and career aspirations [29], increased participation [38], increased engagement [39], and increased task performance [40]. We found that as the percent of women increases in small groups, course grades also increase for all students, and that women (but not men) reported more positive perceptions of their group members’ performance. Our results contrast some work that finds negative perceptions arise when groups are composed of women who succeed at male-stereotyped skills, because attributes women are thought to embody do not fit with those required to accomplish group tasks deemed masculine [31,41]. We believe future research across multiple semesters and in multiple STEM disciplines will clarify the strength and generality of our results, and whether the positive impact of increasing the proportion of women in groups is context-specific.

The positive impacts of small group gender ratios may occur through different mechanistic pathways for women and men, who report different experiences in the classroom with respect to instructor interactions [42], high stakes exams [43], and subtle gendered visual cues [28]. For women, attitudes might influence the extent that gender ratios impact performance and perceptions. For example, women may benefit from being a numeric majority through the reduction of stereotype threat, whereby individuals who are members of a group characterized by negative stereotypes underperform when that group membership is emphasized [44]. Similarly, women may require a critical mass of other women before a sufficient sense of belonging will positively influence performance [45]. Previous work suggests women possess higher social intelligence [40], which could benefit group work as the number of women per group increases. While we do not find evidence that female students’ sense of social belonging increases as the gender ratio becomes more female dominated, we do find that only women view the work of their group members more positively, supporting the idea that affective measures increase as the group’s percent women increases.

Our results show that men also benefit from female-dominated groups. We discuss two possible reasons: first, improved group cohesion in female-dominated groups may have reduced barriers to discussion during the problem-solving sessions which resulted in better understanding for all. Alternatively, mixed-gender groups might provide learning benefits to men when they adhere to masculine gender stereotypes, such as asserting opinions, taking leadership roles, and displaying confidence in responses to questions–qualities that mentors and educators sometimes seek to promote among historically underrepresented students and in active learning classrooms. When tasked with assembling a radio, Myaskovsky et al. [38] found that men became more task-oriented within mixed-gender groups as opposed to same-gender groups (see also: [46–48]). Myaskovsky et al. [38] also found that solo women in groups were less talkative than majority women, but solo men were *more* talkative than majority men. Hollingshead and Fraidin [49] found that people working with mixed-gender partners were more likely to assign tasks for their partner based on their partner’s gender stereotype. In this case, mixed-gender pairs reinforced gender stereotypes rather than eliminated them. If taking on gender-stereotyped roles benefits men’s learning, and increased exposure to women creates a larger sense of obligation to take on those roles, then we may expect men to benefit from female-majority groups. Future research will profit from explicit measures of how group dynamics impact learning, with a focus on men.

We may expect different results in other STEM courses, as undergraduate biology is a numerically female-dominated discipline and in the courses we examined, women reported relatively high levels of social belonging. Biology is among the few STEM disciplines in which women are not nationally under-represented [13]. Due to increases over the past thirty years in women's interest in biology as a field of study and continuation in biology from baccalaureate to a doctorate, it is often assumed that biology as a discipline has overcome gender disparities [13]. Though this has been disputed [15], future work will profit from an examination of the function of group gender composition in stereotypically masculine fields (e.g., physics, math) in the context of active learning pedagogy. If women exhibit behaviors that do not adhere to normative prescriptions according to gender stereotypes, future work may find *decreased* peer evaluations with increasing proportions of women in groups [41]. The course we focused on is a biology course taken by non-biology majors, or those who do not intend to pursue a biology degree. Previous work shows that non-biology majors differ from biology majors with respect to incoming knowledge, perceptions, backgrounds, and skills [50]. However, there is no current evidence as to the transferability of this work. Therefore, we encourage science educators to replicate this work on biology students, along with other STEM and pre-health students. We predict the impacts of group dynamics in such high-stakes environments may be much more extensive than we demonstrate here.

Finally, our results may be explained by the positive influence of the active learning classroom [32]. In our study, instructors used active learning pedagogy throughout the semester, which consistently rewarded ongoing preparation and cooperative group work. In contrast, in traditional introductory gateway classes, students generally work alone and their grades rely primarily on high-stakes exams. As active learning environments disproportionately benefit women [51,52], gender ratio manipulations in these active environments may also impact performance outcomes and perceptions.

We are the first to demonstrate the positive effects of women on students’ performance in small groups in active learning courses. From a broad perspective, our results point to the importance of retaining women as it increases course performance for all students regardless of gender, and promotes a more positive attitude in women toward their peers. These results may have significant impacts in male-dominated STEM disciplines where women are a minority, and we suggest this as an avenue for future investigation. Our results also present a practical solution for instructors aiming to create inclusive teaching spaces: when structuring small groups in active learning classrooms, women will benefit by being clustered together rather than spread out evenly. By making small changes to group composition, instructors can have large positive impacts on learning in active courses.

## Supporting information

S1 Appendix Students’ self- and peer evaluation in same or mixed gender groups. (DOCX)

S2 Appendix Students’ sense of social belonging in the classroom environment assessment. (DOCX)

S3 Appendix Data analysis. (PDF)

S1 Data Performance and social belonging dataset. (CSV)

S2 Data Self- and Peer-evaluation midterm dataset. (CSV)

S3 Data Self- and Peer-evaluation final dataset. (CSV)
