# Supplementary material for: Small group gender ratios impact biology class performance and peer evaluations
Source: PLoS One. 2018 Apr 3;13(4):e0195129. doi: 10.1371/journal.pone.0195129 (PMC5882121; doi:10.1371/journal.pone.0195129)
Supplement: S1 Appendix — (DOCX) [file pone.0195129.s001.docx]

**S1 Appendix. Students’ self- and peer evaluation in same or mixed gender groups.**

Please follow the link below to complete a short, ANONYMOUS evaluation of your group members. We hope that you provide honest, constructive feedback to your group members as part of an ongoing effort to improve group functioning. For instance, if you think someone has great ideas but does not speak up enough, let them know in the comments section.

This information will be shared with your peers, but your name will not be attached to your evaluation.

[Name of group member]

This group member regularly shows up to class.

1 = strongly disagree, 2 = disagree, 3 = neutral, 4 = agree, 5 = strongly agree

This group member regularly contributes ideas and suggestions during group discussions.

1 = strongly disagree, 2 = disagree, 3 = neutral, 4 = agree, 5 = strongly agree

This group member exhibits a strong understanding of course material.

1 = strongly disagree, 2 = disagree, 3 = neutral, 4 = agree, 5 = strongly agree

Comments
