# Supplementary material for: Small group gender ratios impact biology class performance and peer evaluations
Source: PLoS One. 2018 Apr 3;13(4):e0195129. doi: 10.1371/journal.pone.0195129 (PMC5882121; doi:10.1371/journal.pone.0195129)
Supplement: S2 Appendix — (DOCX) [file pone.0195129.s002.docx]

**S2 Appendix. Students’ sense of social belonging in the classroom environment assessment.**

Please rank each of the following according to what extent you agree or disagree that:

Students in the class try to help one another understand course material (e.g. sharing lecture notes when absent)

1 = strongly disagree, 2 = disagree, 3 = neutral, 4 = agree, 5 = strongly agree

Students in the class consider themselves as part of a community.

1 = strongly disagree, 2 = disagree, 3 = neutral, 4 = agree, 5 = strongly agree

I am comfortable making a comment or asking a question during class discussions.

1 = strongly disagree, 2 = disagree, 3 = neutral, 4 = agree, 5 = strongly agree

University of Minnesota demonstrates a strong institutional commitment to diversity.

1 = strongly disagree, 2 = disagree, 3 = neutral, 4 = agree, 5 = strongly agree
