# Supplementary material for: Small group gender ratios impact biology class performance and peer evaluations
Source: PLoS One. 2018 Apr 3;13(4):e0195129. doi: 10.1371/journal.pone.0195129 (PMC5882121; doi:10.1371/journal.pone.0195129)

Sullivan, Ballen & Cotner (2018) Data Analysis

Here we present the code and data needed to produce all analyses and figures in Sullivan, Ballen & Cotner (2018) "The positive power of women: Small group gender ratios in active learning courses impact performance and peer evaluations".

Please contact Lauren Sullivan (lsulliva@umn.edu) for questions relating to this code and data.

Input Data and R packages

```
rm(list=ls())
setwd("~/Dropbox/Peer evaluation gender Lauren and Cissy/R analysis/FINAL_cleanedcode")

library(ggplot2)
library(plyr)
library(lme4)
library(lmerTest)
library(rmarkdown)


#PERFORMANCE DATA & SOCIAL BELONGING DATA
perfdat <- read.csv("performance_belonging.csv", header=TRUE)


#PEER EVALUATION DATA
evaldat_M <- read.csv("peer_evaluation_midterm.csv", header=TRUE)
evaldat_F <- read.csv("peer_evaluation_final.csv", header=TRUE)
```

Performance Analysis and Figure 2

```
#Performance Analysis (Table 1a)
test1 <- lmer(Z_Course ~ SEX + pct_female + COMP_ACT_SCORE
              + (1|Course/Lecture_section/Group), data=perfdat, REML=FALSE)

#summary(test1)


#Figure 2
ggplot(perfdat, aes(x=pct_female, y=Z_Course))+
    geom_point()+
    theme_bw()+theme(aspect.ratio=1)+
    geom_smooth(method='lm', se=FALSE)+
    labs(x="\n\n% Female in Group",y="\nRelative Course Grade (z-score)", title="")
```

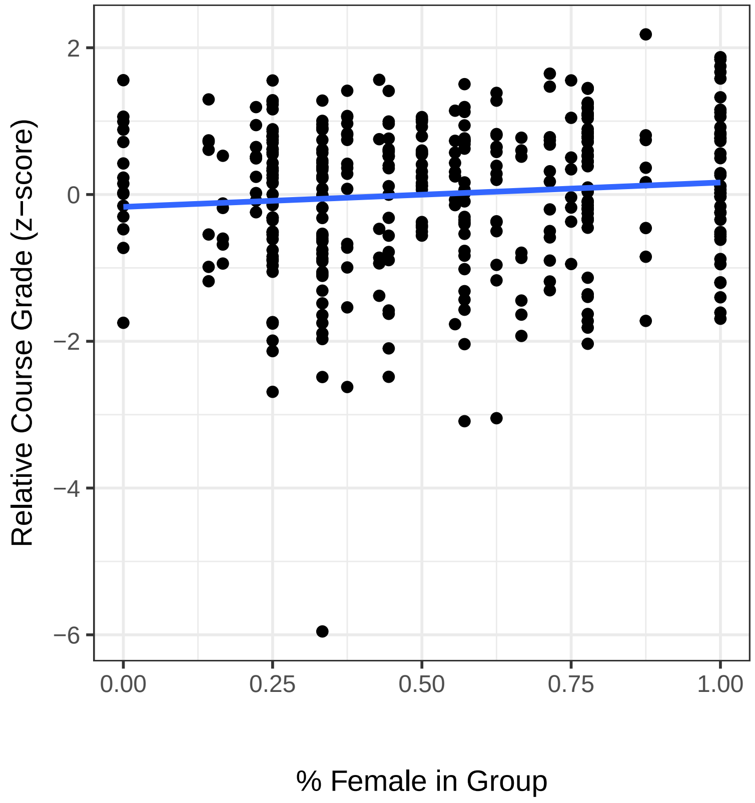

Social Belonging Analysis and Figure 3

```
#Social Belonging (Table 1b)
test2 <- lmer(inclusivity ~ SEX + pct_female + (1|Course/Lecture_section/Group),
              data=perfdat, REML=FALSE)
#summary(test2)


#Figure 3
stats <- ddply(perfdat, .(SEX), summarise, mean_inclus = mean(inclusivity, na.rm=TRUE),
               se_inclus = sd(inclusivity, na.rm=TRUE)/sqrt(length(inclusivity)) )

ggplot(perfdat, aes(x=SEX, y=inclusivity))+
    geom_jitter( width=.02, colour="gray20", alpha=1/3)+
    geom_errorbar(data=stats, mapping=aes(x=SEX,y=mean_inclus, ymin=mean_inclus-se_inclus,
                 ymax=mean_inclus+se_inclus), colour="Blue", lwd=1, width=.05)+
    geom_point(data=stats, aes(x=SEX, y=mean_inclus),
               colour="Blue", size=1, pch=21, stroke=1.5)+
    theme_bw()+
    theme(aspect.ratio=1)+
    labs(x="Student Gender",y="Sense of Social Belonging", title="")+
    scale_x_discrete(labels=c("F" = "Female", "M" = "Male"))+
    ylim(c(1,5))
```

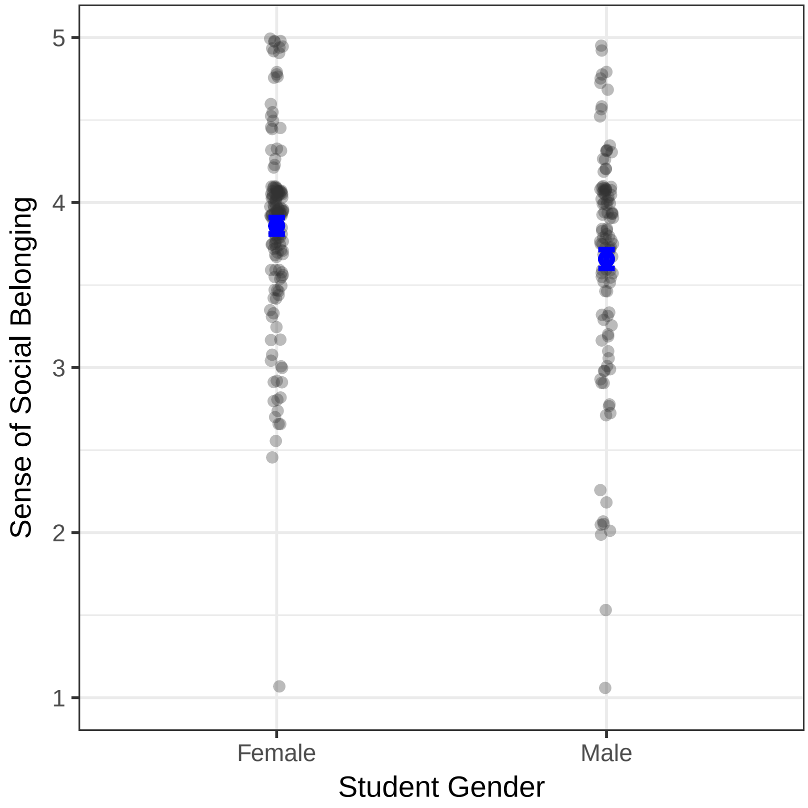

Peer Evaluation Analysis and Figure 4

```
evaldat_M$self <- as.factor(as.character(evaldat_M$self))
evaldat_F$self <- as.factor(as.character(evaldat_F$self))

#Midterm Peer Evaluation
test3M <- lmer(overallmean ~ pct_female + evaluator_SEX +  self  +
                 (1|Lecture_section/Group), data=evaldat_M,REML=FALSE)
#summary(test3M)

#Final Peer Evaluation - Full Model (Table 1c)
test3F <- lmer(overallmean ~ pct_female + evaluator_SEX + self  +
                (1|Lecture_section/Group), data=evaldat_F, REML=FALSE)
#summary(test3F)

#Final Peer Evaluation - Female Only Peer Model (Table 1d)
test3Fa <- lmer(overallmean ~  pct_female   + (1|Lecture_section/Group),
               data=subset(evaldat_F, self==0 & evaluator_SEX=="F"), REML=FALSE)
#summary(test3Fa)

#Final Peer Evaluation - Female Only Self Model (Table 1e)
test3Fb <- lmer(overallmean ~  pct_female   + (1|Lecture_section/Group),
               data=subset(evaldat_F, self==1 & evaluator_SEX=="F"), REML=FALSE)
#summary(test3Fb)
```

```r
#Figure 4
ggplot(evaldat_F, aes(x=pct_female, y=overallmean, shape=self, colour=evaluator_SEX,
                      linetype=self))+
    geom_point(size=2.5)+
    theme_bw()+
    theme(aspect.ratio=1)+
    scale_color_manual(values=c("#009E73", "#e79f00"))+
    geom_smooth(method='lm', se=FALSE)+
    labs(x="\n\n% Female in Group",y="Evaluation Score", title="")+
    theme(legend.position="none")
```

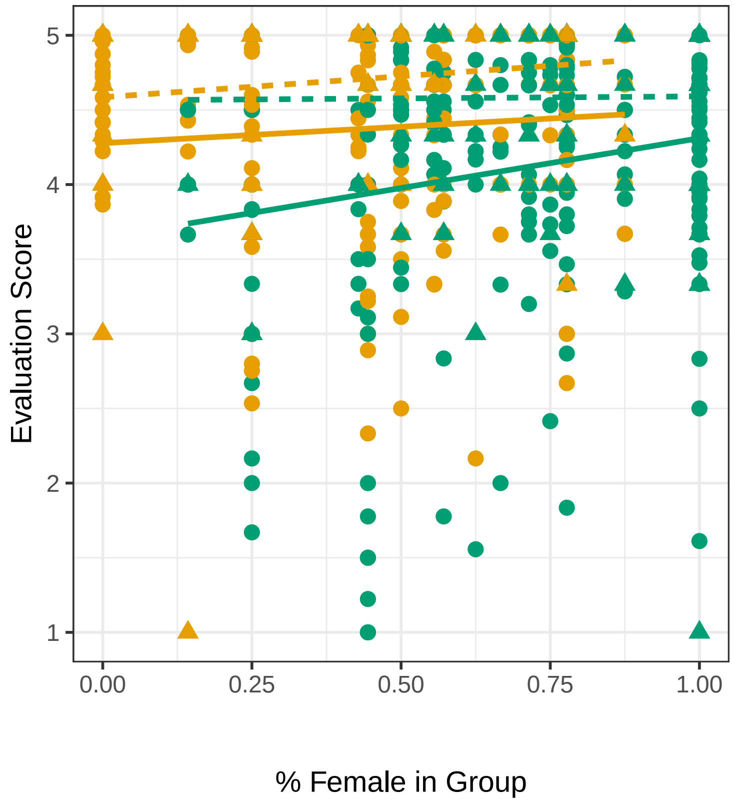

Supplement: S3 Appendix — (PDF) [file pone.0195129.s003.pdf]
